# Evaluation of lymphotoxin-alpha in pterygium and diagnostic value in active and inactive pterygium states

**DOI:** 10.1038/s41598-024-52382-z

**Published:** 2024-01-22

**Authors:** Guoli Lan, Xie Fang, Yanlin Zhong, Shunrong Luo, Xianwen Xiao, Zhiwen Xie, Lianghuan Luo, Yiqiu Zhang, Hanqiao Li, Yuan Lin, Huping Wu

**Affiliations:** 1 Xiamen Eye Center and Eye Institute of Xiamen University, Xiamen, China; 2 Xiamen Clinical Research Center for Eye Diseases, Xiamen, Fujian China; 3 Xiamen Key Laboratory of Ophthalmology, Xiamen, Fujian China; 4 Fujian Key Laboratory of Corneal and Ocular Surface Diseases, Xiamen, Fujian China; 5 Xiamen Key Laboratory of Corneal and Ocular Surface Diseases, Xiamen, Fujian China; 6 Translational Medicine Institute of Xiamen Eye Center of Xiamen University, Xiamen, Fujian China

**Keywords:** Diseases, Signs and symptoms

## Abstract

To explore the correlation between tear LT-a, pterygium status, and dry eye indicators. We established a diagnostic model to evaluate active pterygium. A retrospective study was conducted between June 2021 and June 2023 on 172 patients, comprising 108 men and 64 women. The study analyzed LT-a and various ocular parameters in all participants. The data was collected using Excel software and analyzed using SPSS 25.0 statistical software and Medcalc. We made a nomogram diagnostic model to different diagnosed the state of pterygium. This study found that pterygium has progressive eye surface damage during the active state. There was no significant difference in dry eye indicators between the two groups. However, the concentration of LT-a in the active group was significantly lower than that in the inactive group (*P* < 0.001). We observed that increased pterygium grade corresponded to a worse ocular surface condition. In addition, LT-a was significantly positively correlated with disease duration, but negatively correlated with age, pterygium size, active pterygium state, and LLT value. The optimal intercept value for evaluating active pterygium in Lt-a was ≤ 0.49 dg/ml. We screened three variables for evaluating active pterygium through Single and Multiple regression analysis: LT-a grading, pterygium size, and congestion score. Finally, we made a reliable diagnostic nomogram model. Pterygium development triggers immune inflammation. Our model based on LT-a identifies active pterygium for personalized treatment options and new research directions.

## Introduction

Pterygium is a frequently encountered eye surface disease that presents as a wing-shaped thickening of the conjunctiva that extends over the corneal limbus. Its underlying causes are multifactorial, involving abnormalities in corneal limbal stem cells, epithelial metaplasia, active fibrous vascular tissue, and inflammation, ultimately destroying the Bowman layer at the invasive tip of the pterygium^1–4^. Research has demonstrated that pterygium can disrupt eye surface homeostasis and result in chronic inflammation and dry eye symptoms^5^. In addition, chronic irritation, inflammation, or viral infections impact pterygium growth. The leading cause of pterygium appears to be the upregulation of various pro-inflammatory cytokines, growth factors, and matrix metalloproteinases^6^. Characterizing pterygium requires evaluating fibrosis, angiogenesis, inflammation, and extracellular matrix remodeling^7^. During the active stage of a pterygium, it tends to stimulate and grow, resulting in a relatively severe inflammatory reaction and increased local tissue congestion. It's important to note that if surgery is performed during this stage, there is a heightened risk of recurrence and a more severe postoperative reaction. For this reason, removing the pterygium during the inactive state is generally recommended to minimize the risk of complications and recurrence^8^. Pterygium is a benign growth of fibrous vascular tissue known to be a proliferative disease with a propensity to grow and recur^9^. There are varying opinions on how to manage postoperative ocular surface recovery and recurrence of pterygium, including drug intervention^10^ and surgical techniques^11^ that cause minimal damage to the ocular surface. Nevertheless, there is currently a need for a straightforward and efficient diagnostic tool in clinical practice to ascertain pterygium's ocular surface inflammatory status^12^.

Lymphotoxin a (LT-a) is a member of the tumor necrosis factor (TNF) superfamily and a multifunctional cytokine^13^. Various cells, including CD4 + T helper 1, CD8+, natural killer, B cells, and macrophages, express LTα, which can mediate inflammation, immune stimuli, and antiviral responses and has been associated with inflammation^14^. LT-a contributes to the function of lymphatic vessels and promotes the growth of new lymphatic vessels during inflammation. Additionally, LT-a plays a significant role in the development and proper functioning of the immune system^15^. This involves developing, organizing, and maintaining lymphatic organs, microenvironment, and inflammation-induced lymphangiogenesis^16^. Research indicates that LT-a is an essential factor in regulating the activation of the NF-κB signaling pathway^17^. By stimulating the expression of LT-a and LT-a-R in chondrocytes through the pro-inflammatory cytokine IL-1β, it is possible to modulate the proteins that control inflammation, matrix degradation, and apoptosis of human chondrocytes in vitro^18^. Haiyan Chen et al. discovered increased levels of various tear protein markers in individuals with dry eye syndrome featuring high LT-a levels^19^. In addition, research conducted by Haozhe Yu et al. indicated significant interactions between IL-6R, LT-a, and IL-13^20^. LT-a may be one of the mechanisms causing an immune inflammatory response on the ocular surface during the development of pterygium. Understanding the role of LT-a can help us comprehend the relationship between pterygium and the maintenance of ocular surface homeostasis.

The detection of LT-a can be crucial in preventing symptoms resulting from abnormal ocular surface status of pterygium, which could impact the assessment of its inactive and active. This research focuses on analyzing the LT-a of pterygium in patients and identifying the factors that influence the differentiation between its inactive and active states. By closely monitoring LT-a, we can accurately evaluate the condition of pterygium, gauge the effectiveness of emerging drug therapies, determine the necessity for further surgical intervention, and track postoperative progress. This approach will allow for personalized treatment management plans for each patient.

## Methods

The experimental protocol was established according to the ethical guidelines of the Helsinki Declaration. The studies involving human participants were reviewed and approved by the Human Ethics Committee of Xiamen University affiliated Xiamen Eye Center. The patients/participants provided their written informed consent to participate in this study.

From June 2021 to June 2023, the Xiamen Eye Center conducted a study with 172 patients, consisting of 108 men and 64 women. The study did not include participants who had undergone pterygium resection or had a history of ocular surface diseases that caused dry eye syndrome. The ophthalmologist utilized non-invasive techniques such as LT-a, non-invasive tear break-up time (NIBUT), Tear meniscus height (TMH), meibomian gland (MG) absence score, and lipid layer thickness (LLT) to evaluate the patients. In addition, a slit-lamp camera was used to capture the patients' photographs to determine the pterygium's size and thickness. All tests were conducted in a single examination room by the same ophthalmologist, and patients were asked about their dry eye symptoms history.

### LT-a

**Figure 1:**
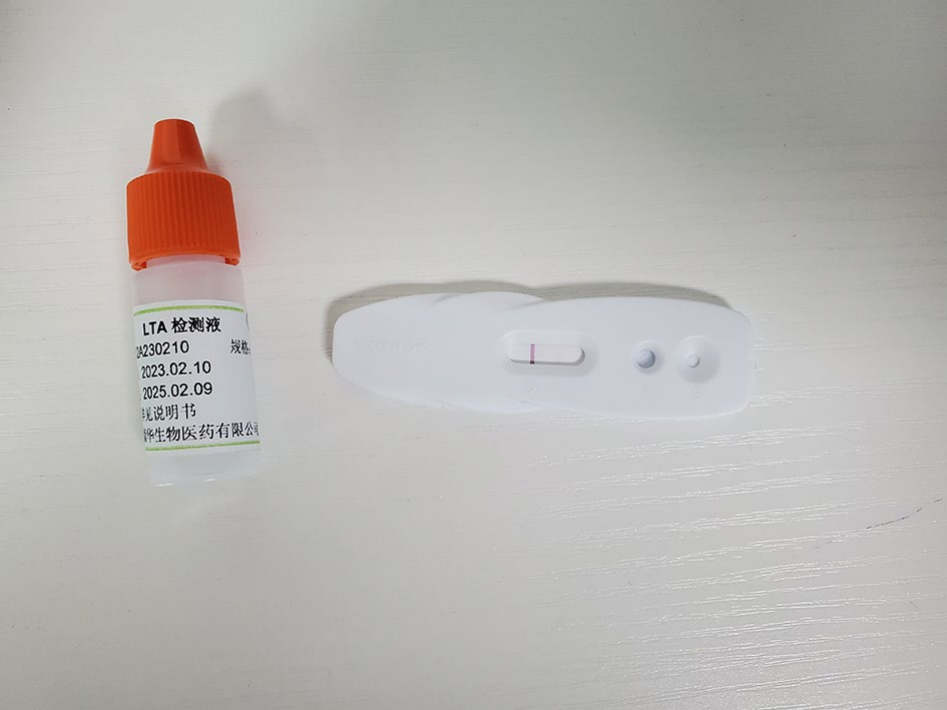
LT-a reagent testing card. The detection card has three areas: the test area containing antibody A, the quality control area containing antibody C, and the sample area with tear drops, where antibody B labeled with red nanospheres, is present. During testing, the sample undergoes chromatographic effects.

According to the complaint of the volunteers, the disposable capillary tear collector was used under the tear lamp to collect one drop of tear tears from the pterygium eye. The collected tears were dropped into the sample area of the LT-a test card (Guangdong Shengze Kanghua Biomedical Co., LTD.). The LT-a test reagent was dropped into the reagent area of the test card, and then the name of the volunteers and the sampling time were recorded on the LT-a test card and left for 10 min. Place the card analyzer and read the parameters (including qualitative results and LT-a concentration). Regardless of whether the antigen is present in the sample, the red nanosphere labeled antibody B is combined with antibody C located in the quality control region to form a red band to determine whether the chromatography process is expected (Fig. 1). The immunochromatography will show qualitative results and LT-a concentration at the end of testing.

### TMH

The TMH was evaluated in a dark room using a corneal topography 5 M corneal camera (Oculus GmbH, Germany). The patient was asked to focus on the fixed target and project a disc of 22 rings onto the corneal surface. Pictures of the lower tear film meniscus were collected after 5 s of blinking, and TMH values were measured with an integrated ruler. All measurements were repeated three times, and their values were recorded.

### NIBUT

In the Non-Invasive Break-Up Time (NIBUT) evaluation, the patient was comfortably positioned in front of a corneal topographer with appropriate chin support. A dorsal disc featuring 22 red concentric circles was projected onto the patient's eye, and they were instructed to blink twice while focusing on the center point. The NIBUT value and pertinent information regarding the tear film break-up size were calculated and presented on the screen.

### Conjunctival congestion score

0 points: no congestion, 1 point: single vessel congestion, 2 points: mild diffuse congestion, 3 points: severe local congestion, 4 points: severe diffuse congestion.

### Meibomian gland loss

Non-contact-type infrared imaging. As mentioned above, the patient sat in front of the 5M anterior corneal camera using the Meibo-Scan procedure and scored: 0, no deletion, 1, < 1/3 gland loss; 2, > 1/3 but < 2/3 gland loss; 3, >2/3 gland loss. Each eye was scored between 0 and 4 and was scored for both eyelids.

### Lipid layer thickness

Mean LLT measurements were obtained using a lipid view interferometer (Tears Science, Inc., Morrisville, North Carolina, USA). Briefly, the patient was instructed to remain fixed to the camera, which recorded a 20-s video of a tear film interferometry image. The unit of measurement used is the interferometric color unit (ICU), an index of the LLT corresponding to about 1 nm with an ICU. With LLT greater than 100 nm, the laser map interferometer showed a maximum of 100 nm.

### Corneal fluorescein staining (CFS)

The corneal fluorescein staining (CFS) was evaluated 3 min after the infusion of fluorescein using a slit-lamp microscope illuminated with cobalt blue. The cornea was divided into four quadrants: superior, inferior, nasal, and temporal. Each quadrant was graded from 0 to 3, where 0 means no staining, one is less than 5, 2 is between 5 and 10, and 3 is more than 10. The total sum of the CFS scores ranged from 0 to 12.

### Assessment of the pterygium

The pterygium size was assessed using a Haag-Streit BQ 900 slit lamp. The size, congestion, and vascularity of the horizontal length from the bottom to the corneal tip were measured. The same experienced operator performed all measurements. Pterygium severity can be categorized into four grades, with Grade I indicating a pterygium at the cornea's edge and conjunctiva appearing as a mound with wing-shaped neovascularization. Grade II involves the pterygium head invading between the corneal and pupillary margins. Grade III features the pterygium head near the pupil's edge, and Grade IV involves the pterygium head penetrating the pupil or crossing the pupil area. Adobe Photoshop CS5 was utilized to determine the pterygium's size.

### Distribution of blood vessels and degree of congestion in pterygium

0: The pterygium's body appears to be a thin film with no apparent congestion.; +: The body of the pterygium looks like a thin film but is slightly congested with a light red hue and slender blood vessels; ++: The pterygium's body is thick in appearance with a reddish hue, and its blood vessels are slightly dilated; +++: The body of the pterygium is thick and has a dark reddish hue, with significantly dilated blood vessels.

### Classification of inactive and active states of pterygium

Inactive stage: The head seems flat, whereas the neck and body appear relatively thin. The blood vessels are also less visible. Although the condition does not progress during this stage, it does not resolve independently either. The head looks pale, with few blood vessels and no growth is detected.

Active stage: As it progresses, the affected area swells and bulges outwards with a broad base and multiple thick blood vessels. The body of the growth spreads out in a triangular shape on either side, while the top part rises and extends into the cornea. During this stage, gray-white structures may resemble caps or satellite lesions on the top part of the growth. Additionally, there could be areas where calcium salt deposition or Stocker iron wires have caused denaturation.

### Statistical analysis

The database was collected and established using Excel software, and the statistical analysis was performed using SPSS 25.0 statistical software (SPSS Inc., Chicago 2017, USA). Continuous variables meet the normal distribution, and equal variance is represented by the mean ± standard deviation (SD). Frequency and percentage representation of categorical variables and interpreted by correlation test. The ocular surface test assessed group differences (Mann–Whitney U test/Kruskal–Wallis test). Medcalc analyzed the optimal intercept value of LT-a concentration, and post hoc test (LSD) to compare intra-group differences. Photoshop CS5 and Prism 8 were used for the picture. The probability of active pterygium was estimated using a nomogram. The consensus index (c index) calculated by bootstrapping and the Area Under Curve (AUC) were used to evaluate the discriminative ability. Calibration charts are used to evaluate calibration capabilities. The C-index and AUC values range from 0.5 to 1.0, where 0.5 represents a random probability, and 1.0 represents a perfect fit. Usually, a c index and AUC value greater than 0.7 indicate that this is a reasonable estimate.

## Result

This study included a total of 172 patients with pterygium, including 108 males and 64 females. There was no statistically significant difference in gender between groups (*p* = 0.071) (Table 1). The mean age was 50.59 ± 11.26 years for the inactive pterygium group, 55.98 ± 9.66 years for the active pterygium group (*P* = 0.006). The disease time was 3.25 ± 2.91 years for the inactive pterygium group, 5.18 ± 5.38 years for the active pterygium group (*P* = 0.033). The disease time was 3.25 ± 2.91 years for the inactive pterygium group, 5.18 ± 5.38 years for the active pterygium group (*P* = 0.033). The pterygium size was 1.69 ± 1.31 mm for the inactive pterygium group, 2.69 ± 1.23 mm for the active pterygium group (*P* < 0.001).

**Table 1:** General information on patients, examination, and test results.

	Inactive group	Active group	Z/χ^2^	*P* value
Sex			0.874	0.071‡
Male	17 (26.5%)	47 (73.5%)		
Female	22 (20.3%)	86 (79.7%)		
Age (years)	50.59 ± 11.26	55.98 ± 9.66	− 2.714	0.006†
Disease time (years)	3.25 ± 2.91	5.18 ± 5.38	− 2.140	0.033†
Pterygium size (mm)	1.69 ± 1.31	2.69 ± 1.23	− 4.913	0.001†
Congestion score	1.31 ± 0.69	1.80 ± 0.56	− 4.573	0.001†
LT-a (ng/ml)	2.16 ± 3.82	0.10 ± 0.15	− 5.516	0.001†
TMH (mm)	0.14 ± 0.04	0.15 ± 0.04	− 0.318	0.750†
NIBUT (s)	6.31 ± 3.32	5.95 ± 6.39	− 1.447	0.147†
CFS	0.82 ± 0.68	0.98 ± 0.75	− 3.584	0.001†
MGS (up)	1.36 ± 0.74	1.32 ± 0.73	− 0.243	0.807†
MGS (down)	0.82 ± 0.82	0.86 ± 0.79	− 0.339	0.734†
LLT	66.23 ± 28.20	70.60 ± 25.21	− 1.083	0.278†

*LLT* lipid layer thickness, *NIBUT* non-invasive break-up time, *CFS* corneal fluorescein staining, *MGS* meibomian gland score.

^†^Mann–Whitney U test. ‡χ2 test.

The summary of the test results and statistical analysis is provided in Table 1. The congestion score was 1.31 ± 0.69 score for the inactive pterygium group, 1.80 ± 0.56 score for the active pterygium group. The Inactive pterygium group had a lower congestion score compared to the active group (*P* < 0.001). The LT-a test was 2.16 ± 3.82 ng/ml for the inactive pterygium group, 0.10 ± 0.15 ng/ml for the active pterygium group. The inactive pterygium group had lower congestion scores than the active group (*P* < 0.001). There was no significant difference in the TMH results between the inactive group and the active group (*p* = 0.750). There was no significant difference in the NIBUT between the inactive group and the active group (*p* = 0.147). The CFS was 0.82 ± 0.68 score for the inactive pterygium group, 0.98 ± 0.75 score for the active pterygium group. The active group had a higher CFS than the inactive group (*P* < 0.001). There was no significant difference in the MGS (up/down) between the inactive group and the active group (*p* = 0.807, *P* = 0.734, respectively). There was no significant difference in the LLT between the inactive group and the active group (*p* = 0.278).

The higher grades of pterygium were found to be associated with older age and, longer disease time (*p* = 0.01; Table 2), and a longer growth time (*p* < 0.001; Table 2). Higher-grade pterygium groups were observed to have a larger size and higher consensus score (*P* < 0.001 and *P* < 0.001, respectively; Table 2). The LT-a test was significantly lower in the III grade group than in the other three groups (*p* = 0.026; Table 2). There was no significant difference in the TMH between 4 groups (*p* = 0.069). An increase in pterygium grade was observed to be associated with a significant decrease in NIBUT (*p* < 0.001; Table 2) and a significant increase in the fluorescent staining score (*p* < 0.001; Table 2). The IV grade group was observed the higher MGS (Up/Down) compared to 3 groups (*p* = 0.012 and *P* = 0.009; Table 2). An increase in pterygium grade was associated with a significant increase in LLT (*p* = 0.005; Table 2). The post hoc test results showed that Grade I statistically significant differences in age, disease time, congestion, LT-a , and CFS compared to Grade II, III and IV (*P* < 0.05), while the differences between Grade II and Grade III, Grade II and IV, Grade III and Grade IV were not significant (*P* > 0.05). In addition, Grade I, II and III showed statistically significant differences in NIBUT, MGS (up) and MGS (down) and LLT compared to Grade IV respectively (*P* < 0.05). The difference between Grade I and Grade II, Grade I and III, Grade II and Grade III were not obvious (*P* > 0.05).

**Table 2:** Dry eye examination results for different pterygium grades.

	I	II	III	IV	H/χ^2^	*P* value
Sex					2.675	0.444‡
Male	5	43	4	12		
Female	10	81	6	11		
Age (years)	45.87 ± 9.27	54.77 ± 10.28	57.30 ± 6.30	59.39 ± 8.80	16.238	0.01†
Disease time (years)	2.04 ± 2.57	4.58 ± 4.83	6.70 ± 5.45	6.50 ± 6.02	17.853	0.001†
Pterygium size (mm)	0.90 ± 0.43	2.13 ± 0.82	4.00 ± 0.81	4.63 ± 1.05	88.671	0.001†
Congestion score	0.93 ± 0.45	1.68 ± 0.60	2.00 ± 0.01	2.09 ± 0.51	33.886	0.001†
LT-a (ng/ml)	2.20 ± 5.17	0.42 ± 1.35	0.09 ± 0.24	0.54 ± 1.35	9.286	0.026†
TMH (mm)	0.17 ± 0.05	0.14 ± 0.04	0.16 ± 0.05	0.15 ± 0.03	7.108	0.069†
NIBUT (s)	7.86 ± 3.08	6.52 ± 6.42	5.34 ± 3.56	4.84 ± 2.74	30.051	0.001†
CFS	0.40 ± 0.73	1.21 ± 1.33	1.90 ± 0.99	2.35 ± 1.15	30.384	0.001†
MGS (up)	1.27 ± 0.59	1.25 ± 0.69	1.20 ± 0.78	1.83 ± 0.83	11.011	0.012†
MGS (down)	0.67 ± 0.81	0.81 ± 0.72	0.40 ± 0.51	1.39 ± 1.03	11.653	0.009†
LLT	55.27 ± 30.63	68.44 ± 24.66	75.10 ± 28.57	82.87 ± 23.20	12.783	0.005†

*LLT* lipid layer thickness, *TMH* tear meniscus height, *NIBUT* non-invasive break-up time, *CFS* corneal fluorescein staining, *MGS* meibomian gland score.

^†^Kruskal–Wallis test. ‡χ2 test.

Correlation between the LT-a and the ocular surface Indicators. LT-a was significantly positively correlated with disease time (R = 0.181, *P* = 0.024) and significantly negatively correlated with that, including the age, pterygium Size, and LLT values (R = − 0.166, *P* = 0.029; R = − 0.215, *P* = 0.004; R = − 0.234, *P* = 0.004, respectively; Table 3). In addition, pterygium level and active pterygium were two key parameters when assessing pterygium. The correlation analysis revealed that pterygium level was positively associated with age (R = 0.292, *p* < 0.001; Table 3), duration of the disease (R = 0.208, *p* < 0.05; Table 3), pterygium size (R = 0.718, *P* < 0.001; Table 3), degree of congestion (R = 0.424, *p* < 0.001; Table 3), and the CFS (R = 0.420, *p* < 0.001; Table 3). The correlation analysis revealed that pterygium level was negatively associated with LT-a (R = − 0.215, *P* = 0.004; Table 3). Moreover, active pterygium was positively associated with age (R = 0.207, *p* = 0.006; Table 3), duration of the disease (R = 0.157, *p* = 0.011; Table 3), pterygium size (R = 0.375, *P* < 0.001; Table 3), degree of congestion (R = 0.349, *p* < 0.001; Table 3), and the CFS (R = 0.274, *p* < 0.001; Table 3). Active pterygium was negatively associated with LT-a (R = − 0.421, *P* < 0.001). Furthermore, active pterygium is positively associated with age (R = 0.207, *p* = 0.006; Table 3), duration of the disease (R = 0.157, *P* = 0.011; Table 3), congestion score (R = 0.349, *P* < 0.001), and CFS (R = 0.274, *p* < 0.001; Table 3).

**Table 3:** Statistical results of the correlation between relevant clinical trials in the pterygium group.

	LT-a		Pterygium level		Active pterygium	
	R	*P*	R	*P*	R	*P*
Sex	− 0.063	0.410	0.106	0.164	− 0.071	0.351
Age	− 0.166	0.029	0.292	< 0.001	0.207	0.006
Disease time (years)	0.181	0.024	0.208	< 0.001	0.157	0.011
Pterygium size (mm)	− 0.215	0.004	0.718	< 0.001	0.375	< 0.001
Congestion score	− 0.062	0.412	0.424	< 0.001	0.349	< 0.001
LT-a (ng/ml)	1	–	− 0.215	0.004	− 0.421	< 0.001
TMH (mm)	− 0.129	0.090	− 0.029	0.700	0.024	0.751
NIBUT (s)	− 0.028	0.709	− 0.410	< 0.001	− 0.110	0.148
*CFS*	− 0.060	0.433	0.420	< 0.001	0.274	< 0.001
MGS (up)	0.041	0.586	0.161	0.034	− 0.018	0.808
MGS (down)	0.063	0.409	0.145	0.056	0.025	0.735
LLT	− 0.234	0.001	0.270	< 0.001	0.082	0.280

*LLT* lipid layer thickness, *TMH* tear meniscus height, *NIBUT* non-invasive break-up time, *CFS* corneal fluorescein staining, *MGS* meibomian gland score.

**Figure 2:**
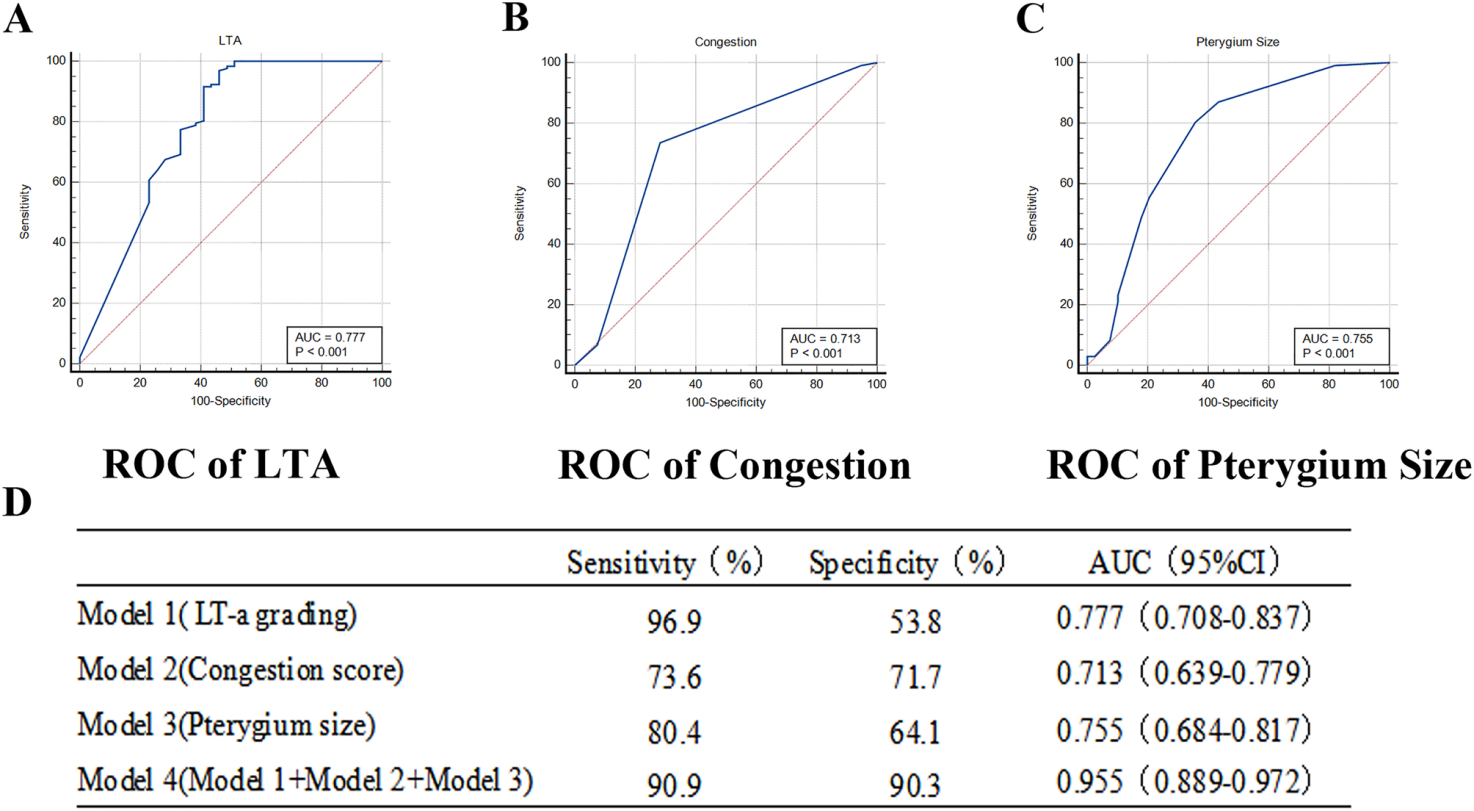
Receiver operating characteristic (ROC) curve analysis for single variables. (**A**) Model 1: LT-a grading. (**B**) Model 2: Congestion score. (**C**) Model 3: Pterygium size. (**D**) The comparison of model. The model composed of individual variables has specific diagnostic efficacy in the active state of pterygium.

Through the results of LT-a detection, we found that when LT-a ≤ 0.49 ng/ml, patients with pterygium were evaluated as in the advanced stage. The ROC curve showed an AUC of 0.777 (95% CI 0.708–0.837) (Fig. 2), a sensitivity of 96.9%, and a specificity of 53.8%. We use the active state as the dependent variable (0 = inactive, 1 = active) to analyze the above statistically significant variables. After single logistic regression analysis results, age, disease time, pterygium size, pterygium level, congestion score, LT-a grading, and CFS were diagnostic factors of active pterygium (Table 4). Based on clinical data and LT-a, through multiple logistic regression analysis (forward: LR), respectively screened out model: LT-a grading (OR 21.687, 95%CI 7.093–66.303) + pterygium size (OR 1.594, 95%CI 1.002–2.537) + congestion score (OR 3.335, 95%CI 1.420–7.836) (Table 4).

**Table 4:** Single and multiple logistic regression analysis of active pterygium.

	Single analysis			Multiple analysis		
	OR	OR (95%CI)	*P*	OR	OR (95%CI)	*P*
Sex	0.707	0.342–1.462	0.349			
Age	1.055	1.016–1.095	0.005			
Disease time (years)	1.131	1.008–1.268	0.035			
Pterygium size (mm)	2.229	1.502–3.306	< 0.001	1.594	1.002–2.537	0.047
Pterygium level	5.061	2.097–12.210	< 0.001			
Congestion score	3.983	2.048–7.749	< 0.001	3.335	1.420–7.836	0.006
LT-a grading (ng/ml)	0.045	0.010–0.200	0.045	21.687	7.093–66.303	< 0.001
TMH (mm)	3.311	0.001–10,268.346	0.770			
NIBUT (s)	0.990	0.936–1.047	0.733			
*CFS*	1.812	1.242–2.641	0.002			
MGS (up)	0.923	0.569–1.497	0.605			
MGS (down)	1.060	0.675–1.663	0.637			
LLT	1.007	0.993–1.021	0.354			

*LLT* lipid layer thickness, *TMH* tear meniscus height, *NIBUT* non-invasive break-up time, *CFS* corneal fluorescein staining, *MGS* meibomian gland score.

**Figure 3:**
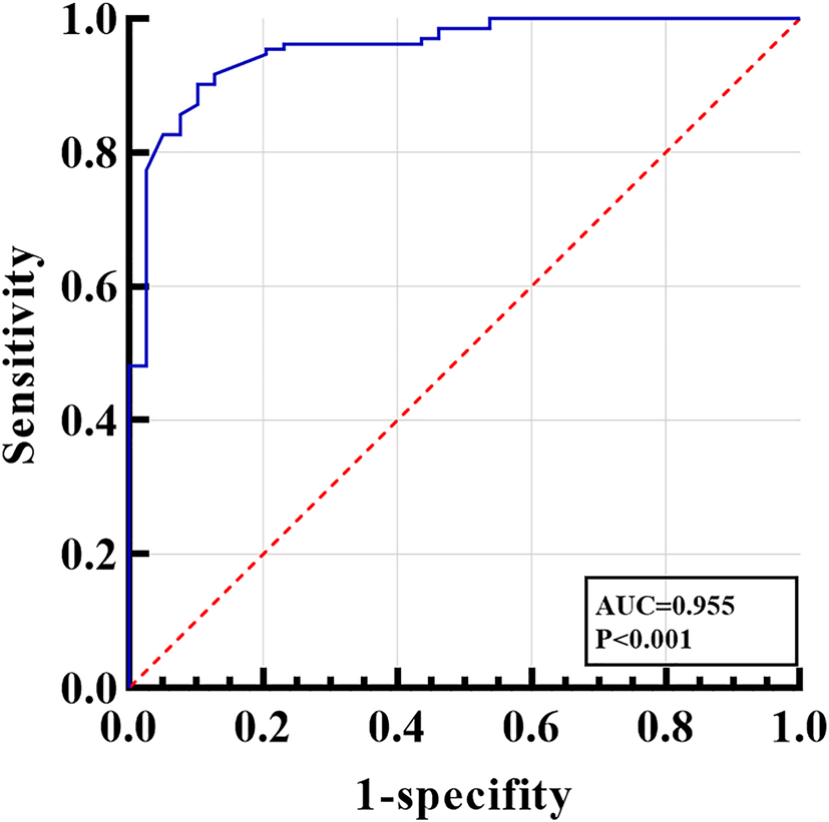
Receiver operating characteristic (ROC) curve analysis for multiple variables. The model 4: LT-a grading + Congestion score + Pterygium size. The diagnostic model fitted after multiple logistic regression has higher diagnostic efficiency.

**Figure 4:**
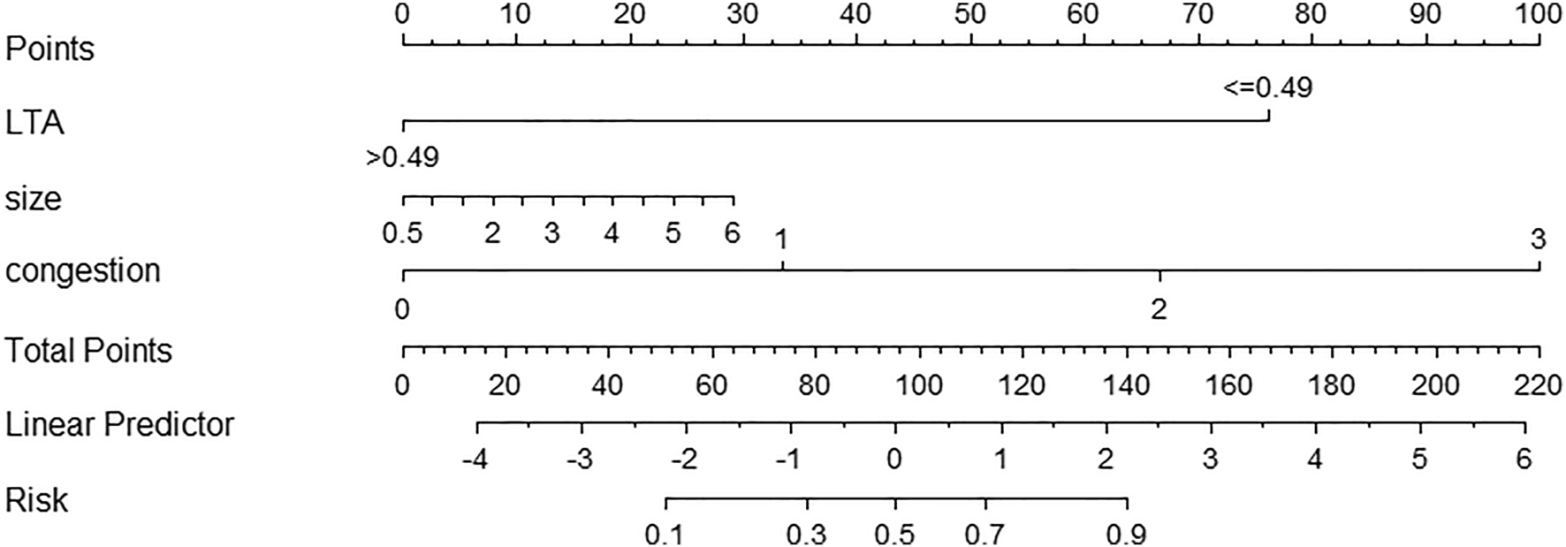
Nomogram prediction model for active pterygium in Model 4. This nomogram model helps predict the diagnosis of an active pterygium patient. The nomogram uses column graphs to plot specific points of individual patients on each variable axis. The sum of these points is then recorded on the total score axis, and a line is drawn down to the predicted probability axis to determine the point at which each variable is received.

The maximum area under the ROC curve value of model 1 (LT-a grading) evaluation was 0.777 (0.708–0.837); the maximum area under the ROC curve value of model 2 (congestion score) was 0.713 (95%CI:0.639–0.779); the maximum area under the ROC curve value of model 3 (pterygium size) was 0.713 (95%CI:0.639–0.779). Model 1, 2, and 3 were both valuable to distinguish active pterygium, while the maximum area under the ROC curve value of model 4, including model 1, model 2, and model 3 variables, was 0.931 (0.889–0.972), which can reflect higher diagnostic value (Figs. 2, 3). We used R language to create a nomogram prediction model for model 4. The nomogram made understanding the diagnostic factors used to identify active pterygium easier. To get the total score, the scores corresponding to the vertical score of each diagnostic factor were added together through score conversion. The predicted result was active (Fig. 4).

**Figure 5:**
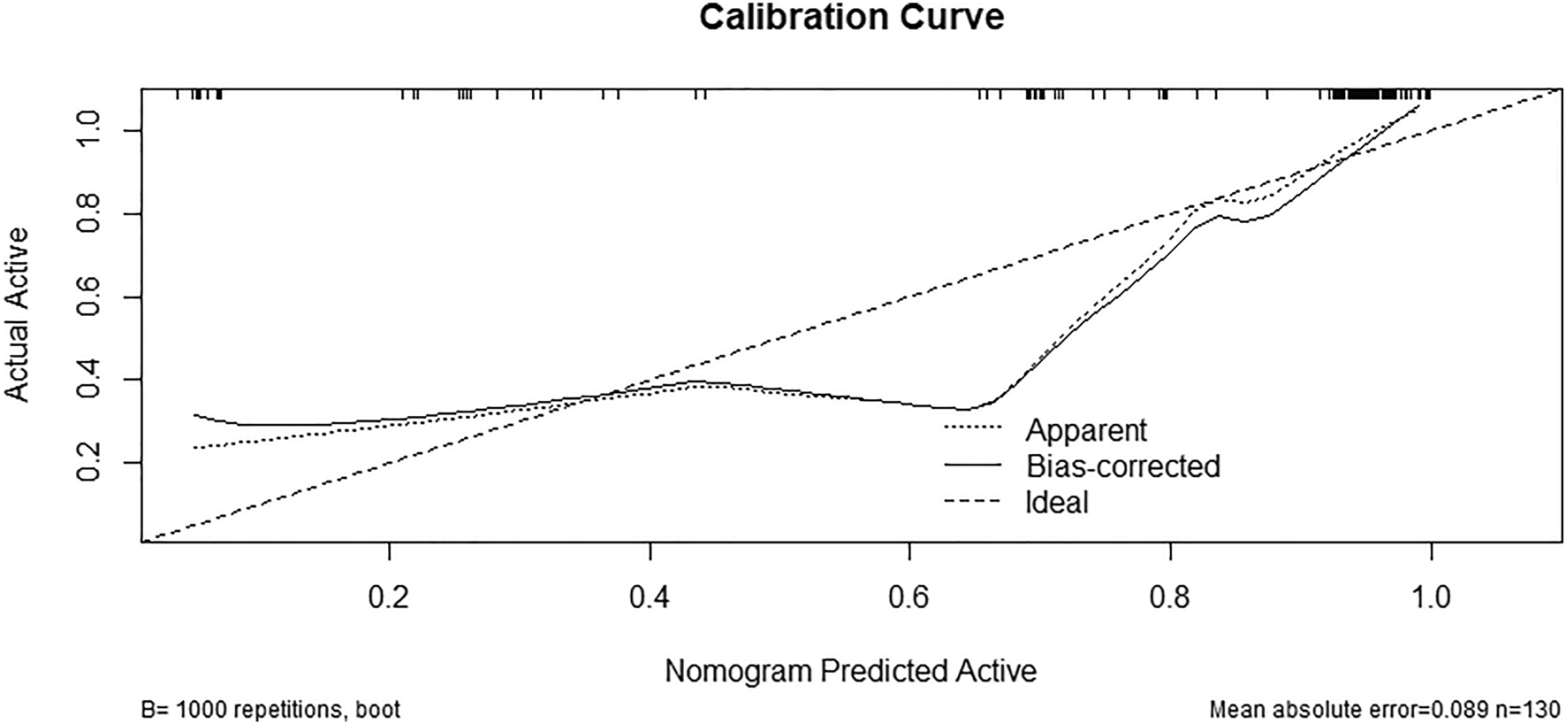
Nomogram model calibration curve for predicting active pterygium. To predict the probability of active pterygium in training, we can use a nomogram and validate it using a calibration curve generated by bootstrap resampling (1000). The calibration curve represents the performance of the nomogram. Ideally, the predictions made by the model should be close to the actual predictions, indicating an accurate prediction of the active rate.

Regarding the model's fit, because of the small sample size, we used the bootstrap method through R language with 1000 repeated extractions to fit the model. The results showed that all models had good prediction ability in the nomogram model fitting results, specifically the prediction of the probability of active pterygium (Fig. 5).

## Discussion

Judging the progression and quiescence of pterygium has always relied on clinical experience. However, hidden inflammations may exist beneath a calm surface. We believe that the progressive development of pterygium can damage the ocular surface and stimulate immune inflammation, which could be a possible reason for pterygium-associated dry eyes. Due to multiple factors affecting the dry eye indicators on the ocular surface^21^, it is not possible to effectively evaluate the tear film instability caused by pterygium. At present, molecular biology research suggests that LT-a detection may be a reliable way to evaluate the immune inflammatory status of the ocular surface and the function of conjunctival goblet cells. Previous clinical studies have suggested that LT-a concentration below 0.9 ng/ml can be diagnosed as dry eye^22^, indicating that the lower the concentration of LT-a, the worse the ocular surface condition. In this study, we analyzed 172 patients with pterygium and divided them into two groups: the inactive group and the active group. We observed that the growth of pterygium affects the ocular surface and found a correlation between the status of pterygium and the concentration of tear LT-a detected by rapid tear detection. We attempted to explain the state of ocular surface immune inflammatory infiltration response during the progression of pterygium and established a reliable nomogram predictive diagnostic model through R language. This contributes to clinical evaluation and management.

Lymphotoxin-α is a member of the TNF superfamily and is produced by various immune cells, including CD4+, Th17, CD8+, NK, and B cells^23^. When tissues experience infection, damage, or inflammation, immune cells such as macrophages and T cells release LT-a^24^. LT-a can combine with LT-β to form trimeric ligands, which bind to LT-β receptors and activate NF-KB and other pathways^25^. Previous research indicates that the double version of LT-α has pro-inflammatory effects on corneal cell lines in vitro^26^. LT-α and LT-β can be expressed through cornea-α1β2 cells and cornea-βR cells, including fibroblasts, epithelial cells, and myeloid cells. The direct interaction between these cells causes the production of inflammatory cytokines and chemokines^27^. Due to the presence of LT-α polymorphism, TNF-α levels increase, which is associated with ocular surface inflammation via one of the potential mechanisms of related inflammatory reactions^28^.

Our study revealed the presence of LT-a in pterygium, indicating that squamous metaplasia of ocular surface epithelial cells caused by immune imbalance may be responsible for the active state of this condition^28,29^. This immune imbalance can decrease mucus secretion and goblet cells, ultimately leading to apoptosis and shedding. Previous research has demonstrated that regulatory T cells (Treg) and protein mediating factors are essential for maintaining immune balance. However, Treg activity is reduced or absent in a dry eye state. LT-a helps maintain ocular surface immune balance through the LT-a TNFR2 Treg axis, which enhances chronic inflammation and is driven by immune responses^30,31^. These immune responses involve both the ocular mucosa and the systemic immune response. Furthermore, Th17 cells secrete IL-17, which promotes the secretion of various vascular endothelial growth factors, resulting in corneal lymphangiogenesis^32^. This leads to immune cell entry into the ocular surface, which hinders Treg activity, further expanding and migrating Th17 and Th1 cells and exacerbating epithelial damage^33^. Our findings suggest that LT-a plays a crucial role in the inflammatory activity mechanism of pterygium.

The diagnostic model for active pterygium combines clinical data and LT-a values to achieve excellent identification and calibration capabilities. Through logistic regression analysis, three variables were selected and included in the nomogram model. LT-a is the most important factor in diagnosing pterygium, followed by congestion score and pterygium size measured by standard deviation on the scale of the nomogram. In the past, accurately determining the active phase in pterygium based on morphology and medical history posed a significant challenge. As a result, our diagnostic model based on LT-a detection can provide valuable assessments in patients with atypical morphology and symptoms. To sum up, we believe that tear molecule testing can provide reference information for the occurrence and development of ocular surface diseases. It can serve as a biomarker for pathological processes and treatment responses.

This study has some limitations as it is a retrospective study carried out in a single center. It may be limited by insufficient sample size and the inability of certain key variables to have significant significance. Additionally, the number of inactive patients is significantly lower than that of active patients, making it difficult to detect all clinical differences. Furthermore, the diagnostic model used in this study may not apply to different regions or races, as the sample size collected from a single center was relatively small. Our future research aims to increase the sample size and discover unique markers or valuable auxiliary tests to achieve precision medicine.

In conclusion, the development of pterygium can have a lasting effect on the health of the ocular surface. The test of LT-a indicates that immune inflammation is provoked by active pterygium formation on the ocular surface. We suggested that the LT-a tear marker can offer insight into goblet cell quantity and performance, indirectly reflecting mucin levels. Mucin is a crucial component of the ocular surface, promoting wetness, lubrication, and barrier function^34^. In cases of dry eye syndrome, these functions are disrupted due to a decrease in the expression of secreted and membrane-associated mucin^35^. Our model based on LT-a has been successfully established to identify active pterygium. By identifying the active phase of pterygium, we aim to provide clinical workers with personalized treatment options and explore new research directions for the pathogenesis of pterygium in the future.

## Data Availability

The datasets generated during and/or analysed during the current study are not publicly available due to individual privacy could be compromised, but are available from the corresponding author on reasonable request.
